# Combination of erythromycin and propranolol for treatment of childhood cyclic vomiting syndrome: a novel regimen 

**Published:** 2015

**Authors:** Mahmood Haghighat, Seyed Mohsen Dehghani, Iraj Shahramian, Mohammad Hadi Imanieh, Alireza Teimouri, Noor Mohammad Noori

**Affiliations:** 1 *Gastroenterohepatology Research Center, Shiraz University of Medical Sciences, Shiraz, Iran*; 2 *Children and Adolescents Health Research Center, Zahedan University of Medical Sciences, Zahedan, Iran*

**Keywords:** Erythromycin, Propranolol, Cyclic Vomiting Syndrome, Therapy

## Abstract

**Aim:**

This study aimed to evaluate the erythromycin efficacy in childhood cyclic vomiting syndrome.

**Background:**

Cyclic vomiting syndrome (CVS) is an unusual cause of episodic emesis in children and erythromycin is an effective treatment.

**Patients and methods:**

In this prospective study, 301 patients with a final diagnosis of CVS enrolled in two separated groups. The first group received erythromycin for 7 days and propranolol for at least 9 months (n=155). The second group was treated with propranolol alone for at least 9 months (n=146). These two groups were compared for response to the treatment and the recurrence of symptoms after treatment completion. Relationship of response, recurrence, and characteristics of the disease was assessed.

**Results:**

Both groups showed a significant difference in terms of response to treatment (P=0.002), however the recurrence after treatment completion had no considerable difference (P=0.563). There was no relationship between CVS characteristics and these two items (response and recurrence).

**Conclusion:**

In our point of view, the addition of erythromycin to standard propranolol treatment can improve the response to treatment, although it has no significant effect on recurrence of CVS symptoms. We suggest the use of erythromycin for 7 days in addition to CVS standard therapy.

## Introduction

 Cyclic Vomiting Syndrome (CVS) is an idiopathic brain – gut disorder with bouts of severe, intractable nonbilius vomiting (1, 2). It is the second most common cause of childhood recurrent vomiting after gastroesophageal reflux (GER) with an estimated prevalence of 1.6% to the ROME III diagnostic criteria for CVS (2). 

There is no importance of propranolol against of erythromycin although in several studies female dominancy has been reported (3-5). CVS is a self-limited process and resolves spontaneously without any therapeutic intervention in the majority of cases considering the patients. This syndrome has documented a link to migraine headache and abdominal migraine (6, 7).

For diagnosis of CVS, fulfilling ROME III criteria is sufficient and in subjects with characteristic clinical manifestations invasive assessments are not essential (2). 

Cyclic vomiting syndrome diagnostic criteria are including all of the following:

1. Stereotypical episodes of vomiting regarding onset (acute) and duration
2. Three or more discrete episodes in the prior year
3. Absence of nausea and vomiting between episodes

Supporting criteria for CVS are as follows: 1-self-limiting nausea, abdominal pain, headache, motion sickness, photophobia and lethargy, as well as 2-accompanying signs of fever, pallor, diarrhea, dehydration, excess salivation and social withdrawal (2).

Although there is no documented effective drug for treatment of CVS in texts, several empirical regimens have been proven in studies that have been based on speculated underlying causes and clinical experiences. For example, in our center as a tertiary referral center, propranolol has been accepted as a treatment for CVS, due to its effectiveness and fewer side effects (2).

The abnormal gastrointestinal motility during asymptomatic and symptomatic period of CVS, including: gastric hypomotility, delayed gastric emptying, gastric dysrhythmia, and small bowel dysmotility has been proved (8-10).

Erythromycin is a macrolide antibiotic with direct acts on motilin receptors and has prokinetic effects. These effects borrowed in using this drug in treatment of CVS in several studies (11-18).

In respect to proven effects of erythromycin on motility disorders and documented motility disorder component for CVS, this study was conducted to evaluate the therapeutic effect of erythromycin in combination with propranolol (standard treatment) in children with CVS. 

## Methods

This prospective clinical trial study was carried out to evaluate the erythromycin efficacy on treatment of patients with cyclic vomiting syndrome. A total of 301 children with an age range from 1 to 18 years old with a final diagnosis of CVS (according to ROMEIII criteria) were treated in pediatric gastrointestinal clinic in Nemazi Hospital, Shiraz, Iran and randomly divided into two groups. The first group received a combination of erythromycin and propranolol (n=155) and the second group was treated with propranolol alone (n=146). Inclusion criteria for the subjects were: Nausea and vomiting with cyclic vomiting syndrome, those who had follow up in outpatient clinics and those aged 1-18 years with vomiting in cyclic recurrence those who fulfilled ROME III criteria for cyclic vomiting. 

The exclusion criteria were as follows: age out of the range between 1-18 years; emerge of organic disease for vomiting such as central nervous system problems, metabolic disorders, and sinusitis; not fulfilling ROME III criteria for cyclic vomiting syndrome and any history of allergic reaction to the drugs consumed.

Cases with drugs complications such as asthma, diabetes, drug hypersensitivity, and cardiovascular problem or unwillingness in every stage of study were excluded.

The first group received erythromycin (20 mg/kg/day PO) for 7 days in addition to propranolol (1 mg/kg/day PO) for 9 months. The second group just received propranolol (1 mg/kg/day PO) for 9 months. The patients were under close observation for 9 months after treatment commenced. The subjects were followed accordance with the determined schedule: in the first day, in the first, second, third, fourth, fifth and sixth months for clinical evaluations

Response was defined as one-month symptom free period after commencing treatment and recurrence was recurring symptoms after 6-month symptom free period. The primary outcome measure was at least 6 months symptom free after commencing the treatment. After completing the data collection, the related information were analyzed in SPSS, v. 20. Histogram for qualitative variables was used along with Pearson, - Chi-square and independent *t*-test. Significance level of 0.05 was considered for the differences. 

## Results

The results of the study showed that in patients who were treated with erythromycin, 58.06% and 41.93% were male and female, respectively. This distribution for percentages of propranolol treatment was 57.53% and 42.46%. Total participants were classified into males and females of 57.8% and 42.19% respectively and this sex gap did not show any significant differences (P=0.509).

The means age of patients in the erythromycin and propranolol groups were 6.08 and 5.88, while for all the patients it was 5.98 years. The results of the independent *t*-test showed no significant difference in age for both erythromycin and propranolol groups (P=0.648). Onset mean age were 3.50 and 3.90 years for groups receiving propranolol and erythromycin respectively while the onset mean age for all participants was 3.74 years. The results showed no significant difference in age for both erythromycin and propranolol groups (P=0.308).  The mean age at diagnosis of propranolol and erythromycin in all patients was 5.89, 6.09 and 5.94 years, respectively (P=0.647).

Table 1, showed the frequency of some clinical symptoms in participants treated with erythromycin and propranolol. The most common symptom for patients who received propranolol, erythromycin and all participants was vomiting with the percentage of 97.94, 96.77 and 97.3, respectively and the second was nausea with 52.73%, 56.77% and 54.8%, respectively. The lowest rate was the sign of constipation (0.00%, 0.64% and 0.33%, respectively). For all of symptoms considered in this study, no significant differences were observed.

**Table 1:** Distribution of clinical symptoms of patients based on two types of treatments

Variable	Propranolol =146(%)	Erythromycin =155 (%)	Total =301(%)
Vomiting	143(97.9)	150(96.7)	293(97.3)
Nausia	77(52.7)	88(56.7)	165(54.8)
Pain	39(26.7)	46(31.5)	85(28.2)
Headache	16(10.9)	23(14.8)	39(12.9)
Diarrhea	15(10.2)	24(15.4)	39(12.9)
Constipation	0(0)	1(0.6)	1(0.33)
Fever	15 (10.2)	17(10.9)	32(10.6)

**Table 2:** Distribution of the underlying factors of CVS in patients

Factor	Propranolol	Erythromycin	Total	P-value
Pervious history of motion sickness	24(16.4%)	19(12.2%)	43(14.2%)	0.192
Family history of motion sickness	7(4.7%)	7(4.5%)	14(4.6%)	0.562
Family history of migraines	67(45.8%)	69(44.5%)	136(45.1%)	0.515
Pervious history of epilepsy	0(0)	1(0.6%)	1(0.33%)	0.666
Family history of epilepsy	2(1.3%)	2(1.2%)	4(1.3%)	0.451

Table 2 showed the results for CVS underlying factors in patients. None of these underlying factors had a significant difference in the frequency between two groups of propranolol and erythromycin. The most common factor was a family history of migraine, and then personal history of motion sickness was placed in the second order. In the comparison of the means duration attack for the propranolol (2.18±1.50) and erythromycin (2.20±1.70), *t*-test revealed that there was no significant difference (P=0.95). 

The table 3 clearly shows the results that none of CVS periodic attacks variables had a significant difference in duration for patients. The response rates to therapy in patients treated with propranolol and erythromycin were 77.39% and 90.32% respectively.  

**Table 3:** The results of t *t*-test in comparison of CVS periodic attacks’ averages in patients

Variable	Propranolol	Erythromycin	Total	P-value
Duration attack				
	2.18±1.50	2.20±1.70	2.19±1.6	0.95
Duration attack similarity (%)				
	145(99.3)	154(99.3)	299(99.3)	0.736
Duration interval				
	4.90±3.12	4.78±3.06	4.08±3.0	0.743
Duration interval similarity (%)				
	146(100)	154(99.3)	300(99.6)	0.515

The difference in the response rates between the two groups was statistically significant (P=0.002). The recurrence rate in propranolol and erythromycin were 8.21% and 8.38% respectively. Despite the significant response to treatment in both groups, the relapse rate was similar in both groups (P=0.563).

Table 4 displayed the relationship between treatment response and clinical symptoms. All participants without considering the type of treatment, nausea (X^2^= 3.237, P=0.049), diarrheal (X^2^=3.912, P=0.031), and constipation (X^2^= 5.288, P=0.021) showed the relationship with responding to the treatments. 

It means that, by considering these symptoms, the frequency of response to treatment was related with involving this difficulty. In patients treated with erythromycin, this pattern didn’t showed any relationship unless constipation (Pearson X2= 9.394, P=0.002) and when considering propranolol no relationship. 

Mean ages in different times of interest was similar for patients who responded to treatment and those who did not in both types of treatments. Means ages in different times of interest were similar for patients who had recurrence to disease and those who did not in both types of treatment (Table 5).

The means of various times of attacks were compared in patients treated with propranolol and erythromaycin in response position. The results in Table 6 showed that no differences were observed between the means of variables in two options of response for patients treated with propranolol and those treated with erythromycin. 

Means of various times of attacks were compared in patients treated with erythromycin and propranolol in recurrence position. Table 6 also showed that no differences were observed between the means of variables in two options of recurrence for patients treated with propranolol and those treated with erythromaycin. 

**Table 4:** The relationship between responses to treatment in patients with several clinical symptoms

Symptoms		Response drug		P	Response erythromycin		P	Response propranolol		P
		YES(253)	NO(48)		YES(14)	NO(15)		YES(113)	NO(33)	
vomiting	yes	246(84)	47(16.04)	0.787	135(90)	15(10)	0.457	111(77.6)	32(22.4)	0.653
	no	7(87.5)	1(12.5)		5(100)	0(0)		2(66.7)	1(33.3)	
Nausea	yes	113(80.6)	32(19.4)	0.049	78(88.6)	10(11.4)	0.416	55(71.4)	22(28.6)	0.051
	no	120(88.2)	15(19.8)		62(92.5)	5(7.5)		58(84.1)	11(15.9)	
Pain	yes	72(84.7)	13(15.3)	0.846	43(93.5)	3(6.5)	0.388	29(74.4)	10(25.6)	0.596
	no	181(83.8)	35(16.2)		97(89)	12(11)		84(78.5)	23(21.5)	
Headache	yes	33(84.6)	6(15.4)	0.918	21(91.3)	2(8.7)	0.863	12(75)	4(25)	0.808
	no	220(84)	42(16)		119)90.2)	13(9.8)		101(77.7)	29(22.3)	
Diarrheal	yes	37(94.9)	2(5.1)	0.031	23(95.8)	1(4.2)	0.321	14(93.3)	1(6.7)	0.119
	no	216(82.4)	46(17.6)		117(89.3)	14(10.7)		99(75.6)	32(24.4)	
Constipation	yes	0(0)	1(100)	0.021	0(0)	1(100)	0.002	_	_	_
	no	253(84.3)	47(15.7)		140(90.9)	14(9.1)		113(77.4)	33(22.6)	
Fever	yes	26(81.3)	6(18.8)	0.647	15(88.2)	2(11.8)	0.758	11(73.3)	4(26.7)	0.691
	no	227(84.4)	42(15.6)		125(90.6)	13(9.4)		102(77.9)	29(22.1)	

**Table 5:** Comparison of age at various situations in patients treated with propranolol and erythromaycin in response and recurrence

Action to drug	variables	option	Number	Propranolol		p	Number	Erythromycin		p
				Mean	SD			Mean	SD	
Response	age	Yes	113	5.695	3.819	0.264	140	6.043	3.821	0.665
		No	33	6.53	3.566		15	6.5	4.44	
	onset age	Yes	113	3.358	3.282	0.323	140	3.854	3.484	0.592
		No	33	4	3.223		15	4.367	3.824	
	diagnosis age	Yes	113	5.704	3.844	0.271	140	6.043	3.847	0.622
		No	33	6.53	3.566		15	6.567	4.375	
Recurrence	age	Yes	12	7.25	3.25087	0.191	13	7.8462	4.38931	0.087
		No	134	5.7612	3.79737		142	5.9261	3.79714	
	onset age	Yes	12	4.2083	2.76716	0.437	13	5.3077	3.83264	0.132
		No	134	3.4403	3.31155		142	3.7746	3.46286	
	diagnosis age	Yes	12	7.25	3.25087	0.195	13	7.8462	4.38931	0.09
		No	134	5.7687	3.81856		142	5.9331	3.81683	

**Table 6:** Comparison of various times of attack in patients treated with Propranolol and Erythromaycin in response and recurrent positions

Action	variables	option	Number	Propranolol		p	Number	Erythromycin		p
				Mean	SD			Mean	SD	
Response	duration attack	Yes	113	2.1681	1.493	0.765	140	2.1714	1.68	0.525
		No	33	2.2576	1.57		15	2.4667	1.95	
	Interval attack	Yes	113	4.9381	3.08	0.809	140	4.75	3.08	0.647
		No	33	4.7879	3.32		15	5.1333	3.02	
	Onset attack	Yes	113	2.4336	0.925	0.145	140	2.4143	0.974	0.577
		No	33	2.697	0.847		15	2.2667	0.961	
Recurrence	duration attack	Yes	12	2.5208	1.90233	0.427	13	2.7115	1.5133	0.259
		No	134	2.1586	1.47171		142	2.1532	1.71426	
	Interval attack	Yes	12	4.1667	2.65718	0.395	13	4.3077	1.84321	0.375
		No	134	4.9701	3.16451		142	4.831	3.15323	
	Onset attack	Yes	12	2.3333	1.07309	0.528	13	1.9231	1.25576	0.168
		No	134	2.5075	0.89901		142	2.4437	0.93418	

## Discussion

CVS is a self-limiting disease and is most common in children. CVS is recurrent episodic attacks of nausea and vomiting with complete resolution of symptoms between attacks. The incidence of CVS is 3.5/100,000 with the prevalence of 2%. 

The cause of CVS is currently unknown, but there may be a link with corticotropin-releasing factor and vasopressin release at the hypothalamic–pituitary level, autonomic dysfunction, disorders of fatty acid, and mitochondrial metabolism with maternal inheritance (19).

 Nowadays, CVS is not a rare condition in children and adolescents (2). There is no specific treatment, nor a simple clinical or biochemical test to establish a diagnosis of CVS. Also, the lack of medical awareness of CVS makes the treatment more challenging (2, 7, 15, 16). Haghighat and Olson recommended that, propranolol is not necessary for evaluation and diagnosis of CVS in cases with typical symptoms and they should be served for atypical presentation and suspicion to other diagnoses that have similar manifestations with CVS (2, 20). Therefore, in the present study we used the ROME III criteria for Diagnosis of CVS and no invasive evaluation was necessary for patients.

In our study, the mean age of symptoms onset and age of diagnosis were lower than other series of studies, such as Haghighat (2) Fleisher (17) and Abu-Aref (1) with the means of onset age of 5, 6.75 and 5.3 years respectively.

But it bears a close resemblance with the study conducted by Hoyt and Stiker that the median age of symptoms commencing 3.8 years (18). The present study revealed no difference between groups in terms of the mean age of symptoms onset and the mean age of diagnosis.

Migraine headache and abdominal migraine have a close link with CVS and this connection resulted by logical reason for some authors that made the treatment of CVS according to the existence of the migraine history (6, 7). 

Because migraine involves nausea and vomiting, there are speculations on similarities in the mechanisms of migraine and CVS (6). Also, changes in brain waves during the vomiting episodes have confirmed that CVS is comparable to migraine (7). The results of our study showed no considerable disparity in terms of migraine history between our groups.

 In respect to propranolol, two groups of patients had no significant difference; Abu-Arefeh and Haghighat have reported similar results (1, 2). 

 However, several authors have reported female preponderance (3-5) and also Vanderhoof reported male dominancy (12).

The patients treated with a combination of erythromycin and propranolol had a statistically higher response to medical therapy compared with propranolol alone.

 Vanderhoof conducted an uncontrolled study on 24 children and showed the effectiveness of erythromycin in the treatment of CVS (12). According to Pavlovic et al., erythromycin led to the disappearance and prevention of vomiting attacks without any adverse effects of the drug, and was suggested as periodical erythromycin for treatment and prevention of CVS (14). Also, Bouaziz et al. showed the efficacy of erythromycin in the treatment of CVS and recommended using this drug in the CVS treatment (16). We did not observe any side effects of erythromycin in patients. Our results supported by other studies (11-16). Our results showed that erythromycin had no considerable influence on recurrence of CVS symptoms, after initial response, which could be due to short half-life of erythromycin and also, it is a multiline receptor agonists that facilitates gastric emptying, relapses the pylorus, induces antral contractions, and has no generalized efficacy (12, 15). Tricyclic antidepressants (TCAs) are commonly used for a variety of functional bowel disorders, including functional abdominal pain, irritable bowel syndrome, and CVS. TCAs are facilitating tools of GABA-ergic neurotransmission and effects on noradrenaline and serotonin nerve pathways. Other antimigraine prophylactic agents include propranolol and topiramate, with reported similar antimigraine efficacy to amitriptyline. Given the benefit of antimigraine and antiepileptic treatments in related disorders such as CVS, these agents are more effective for chronic nausea (21). In migraine-associated CVS and children with CVS, beta adrenoceptor antagonist propranolol has controlled the symptoms and prevented attacks successfully. Anti-migraine drugs that are effective at reducing the number of episodes or severity of migraines include sumatriptan, propranolol and topamax (22). Caron and Broad demonstrated that, when given orally with food at single therapeutic doses, the crystalline formulation of erythromycin ethylsuccinate induces changes in duodenal motility during the fed state. Erythromycin could be a cause to facilitate the empty of stomach and hence reduce vomiting (23, 24).

The results of our study indicate effectiveness and safe effects of erythromycin compared to propranolol. Hence, we suggest the use of Erythromycin in combination to propranolol for treatment of CVS in children. In a conclusion, the age of onset, age at diagnosis, and sex had no effect on response to drugs. We also conclude that there is a significant delay between the onset of symptom and final diagnosis. 

Adding erythromycin to propranolol could increase response to treatment in compared to only propranolol in children with CVS. 

The side effect not increased in using of erythromycin as a treatment. Erythromycin had no impact on recurrence of CVS after responding to treatment.
